# Plexiform Neurofibroma of the Wrist: Imaging Features and When to Suspect Malignancy

**DOI:** 10.1155/2013/493752

**Published:** 2013-04-04

**Authors:** Maria Gosein, Anthony Ameeral, Renee Banfield, Murrie Mosodeen

**Affiliations:** San Fernando General Hospital, Independence Avenue, Paradise Pasture, San Fernando, Trinidad and Tobago

## Abstract

Plexiform neurofibromas are essentially pathognomonic for neurofibromatosis type 1 (NF1), occurring when there is diffuse involvement along a nerve segment and its branches. Transformation into a malignant peripheral nerve sheath tumour (MPNST) is a major cause of mortality in NF1 patients. These tumours are highly aggressive and particularly difficult to diagnose in NF1 patients due to the clinical overlap between benign and malignant lesions. We present a case of a plexiform neurofibroma and discuss the typical imaging characteristics on ultrasound, CT, and MRI, including the target sign and continuity with the parent nerve. Certain imaging features should raise suspicion for malignancy however, these modalities may not always reliably differentiate between benign and malignant lesions. Recent studies show a very high negative predictive value for FDG-PET making it quite useful in excluding malignancy. In positive scans, PET/CT aids in guiding biopsy to the most metabolically active area of the tumour.

## 1. Introduction

Neurofibromatosis type 1 (NF1) is an autosomal-dominant disorder affecting 1 in 3500 individuals [1]. Plexiform neurofibromas are essentially pathognomonic for NF1 occurring with diffuse involvement along a nerve segment and its branches, giving a “bag of worms” appearance [2]. The estimated prevalence of transformation to a malignant peripheral nerve sheath tumour (MPNST) is approximately 5% [2]. Early diagnosis is crucial for effective treatment of MPNSTs but they are particularly difficult to diagnose in NF1 patients due to clinical overlap between benign and malignant lesions [3]. We present a case of a plexiform neurofibroma and discuss the features that should raise suspicion for malignant change.

## 2. Case History

**Figure 1:**
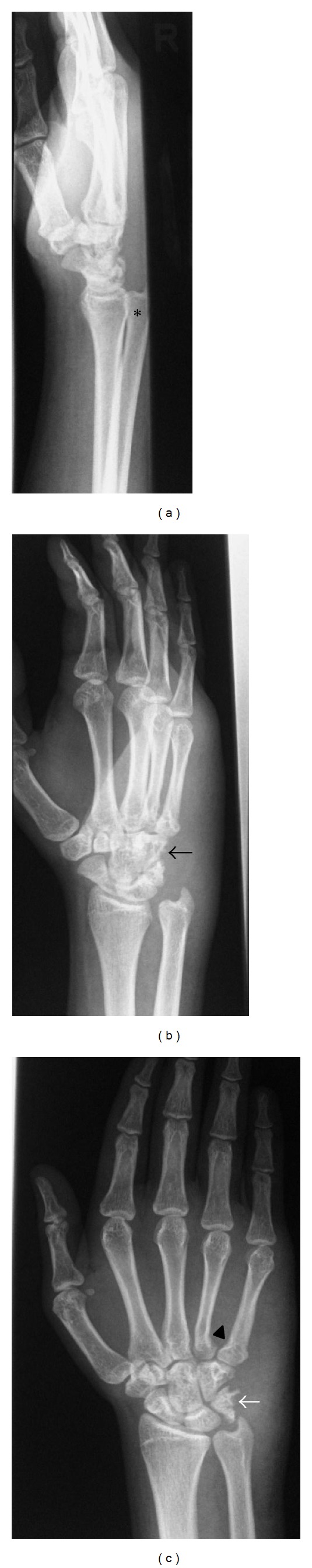
X-ray of the right wrist: (a) lateral, (b) oblique, and (c) AP—soft tissue swelling overlying ulnar aspect of wrist with remodeling of triquetrum (white arrow), hamate (black arrow), and 4th proximal metacarpal (arrowhead). *posterior subluxation of ulna.

A teenage male patient presented with progressive swelling of the right wrist for three years and pain for two days following blunt minor trauma. He reported a four-year-old injury to the wrist with a ball and repeated blunt trauma to the wrist since. No significant past medical or surgical history was elicited. Physical examination revealed a swollen and slightly tender ulnar aspect of the wrist with slight decrease in range of motion. The ulnar nerve was intact clinically with normal sensation and grip strength. Baseline blood results were normal. X-ray (Figure 1) showed soft tissue swelling on the ulna aspect of the wrist with triquetrum remodeling and ulna subluxation.

**Figure 2:**
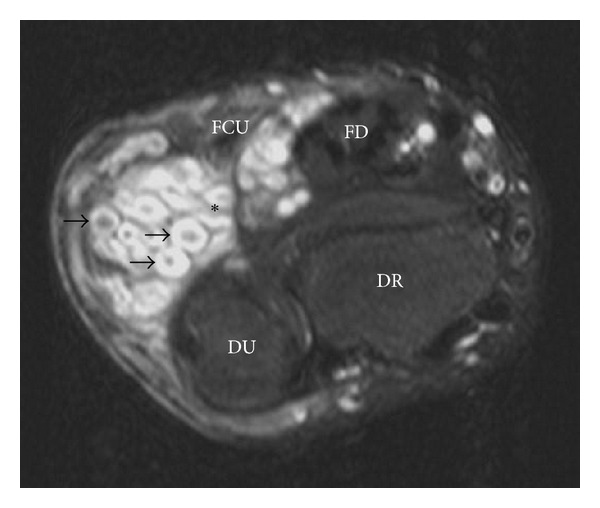
Axial T2-weighted wrist MRI with fat suppression—multilobulated right wrist mass (anterior/ulnar aspect). Individual lesions (arrows) show target signs: rim of high-signal intensity surrounding central low-signal intensity. *usual location of ulnar nerve. DR: distal radius; DU: distal ulna; FD: flexor digitorum longus; FCU: flexor carpi ulnaris.

**Figure 3:**
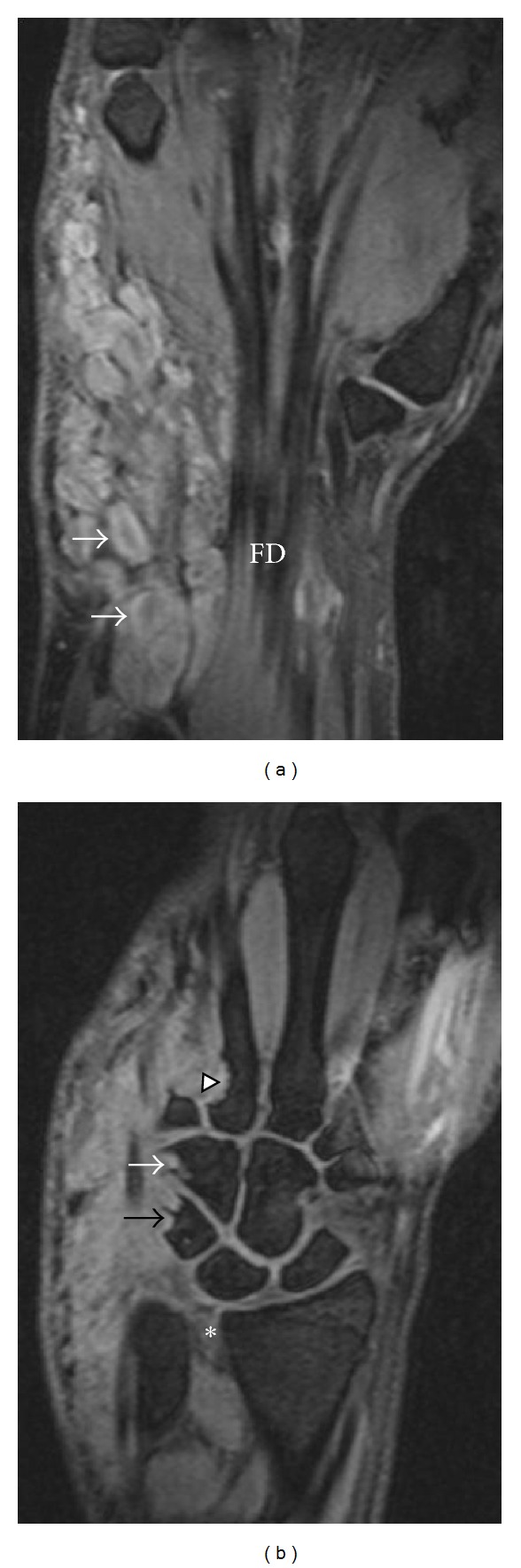
Coronal T2-weighted wrist MRI with fat suppression—(3a) anterior section: multilobulated mass abutting flexor digitorum longus (FD). Target signs (white arrows). (3b) Posterior section: remodeling of triquetrum (black arrow), hamate (white arrow), and proximal metacarpal (arrowhead). *distal radioulnar joint widening.

**Figure 4:**
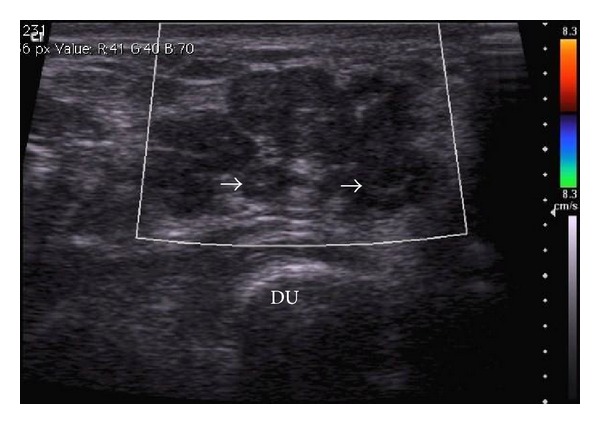
Ultrasound of the wrist—conglomerate multilobulated lesions with target appearance (white arrows): central hyperechogenicity with hypoechoic periphery. No vascularity noted on colour Doppler. DU: distal ulna.

**Figure 5:**
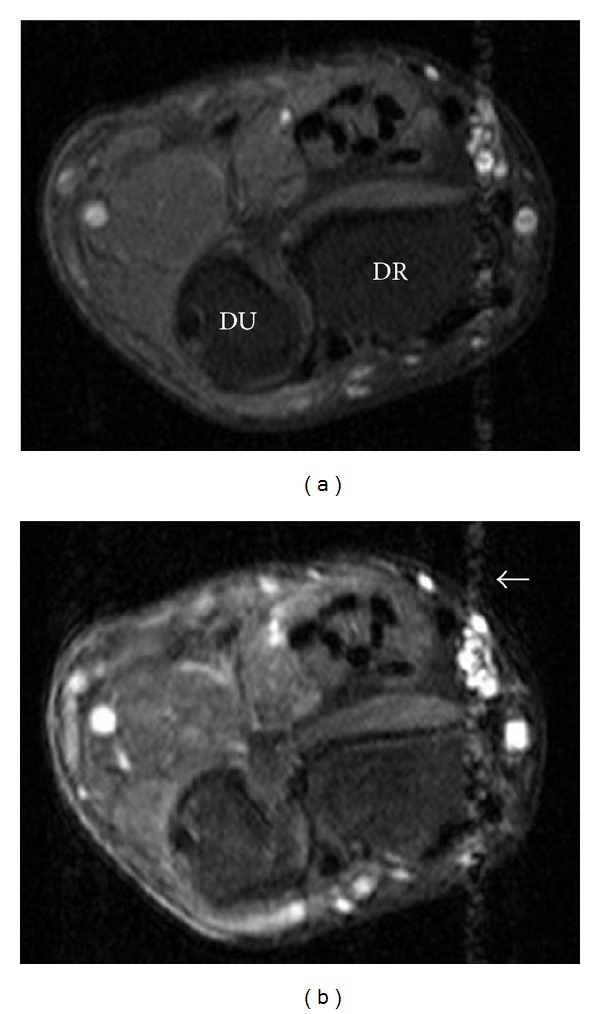
Axial T1-weighted wrist MRI with fat suppression—(5a) precontrast, (5b) postcontrast: the majority of the lesion (seen more clearly on T2-weighted image; Figure 2) showed no significant enhancement. Pulsation artifact from radial artery (white arrow). DR: distal radius; DU: distal ulna.

Wrist MRI (Figure 2) revealed a multilobulated mass (11.0 cm × 4.2 cm × 3.4 cm) along the distal forearm and wrist with adjacent carpal and metacarpal remodeling (Figure 3). This mass extended along the ulnar nerve distribution, and individual lesions showed low central signal intensity with high T2 signal peripherally (“target sign”: Figures 2 and 3). There was minimal enhancement on contrast MRI and absence of flow on colour Doppler ultrasound (Figures 4 and 5). Ulnar nerve continuity was confirmed at ultrasound, consistent with a plexiform neurofibroma. Borders appeared well defined apart from areas adjacent to bony remodeling.

At follow-up visits he reported slight increases in wrist swelling and intermittent pain following trauma; however, sensation and motion remained intact. No other stigmata of neurofibromatosis were found on examination. The patient and his family opted for surgery due to the intermittent pain, swelling, and uncertainty of malignancy. A lobulated tumour was found involving the ulnar nerve and its branches, which could not be removed completely due to its intimate nerve relationship. The majority of the mass was removed, preserving deep and main nerve branches. Postoperatively, the patient had no motor deficits but reported tingling along the ulnar nerve distribution, which subsequently resolved. Histology confirmed a plexiform neurofibroma with no evidence of malignant change. Plexiform neurofibromas are essentially pathognomonic for NF1, rarely occurring outside of this neurocutaneous syndrome [4]. Additional signs of NF1, however, often manifest over time on follow-up examination. This patient will be monitored annually with thorough clinical examination.

## 3. Discussion

The major cause of mortality in NF1 patients is transformation of plexiform neurofibromas into MPNSTs [1]. The five-year survival for MPNSTs is 16% for NF1 patients compared with 53% for non-NF1 patients [3]. The most effective treatment is early diagnosis and surgery; however, diagnosis in NF1 is complicated by clinical overlap between benign and malignant lesions [3]. MPNSTs more often present with pain, rapid growth, and neurologic deficits; however, rapid growth may also be seen in pregnancy and puberty [2]. Similarly, motor and sensory deficits and pain, particularly following trauma [3], can occur in benign lesions; hence, tissue diagnosis is usually necessary. This is complicated by the fact that a malignant component can be missed on biopsy as it may represent a small portion of a large plexiform tumour [3].

Ultrasound, CT, and MRI can be useful in the noninvasive diagnosis and characterization of nerve sheath tumours. On ultrasound, most peripheral nerve sheath tumours (PNSTs) are hypoechoic with posterior acoustic enhancement, sometimes mimicking cystic lesions; however, peripheral nerve continuity is diagnostic [5]. The target appearance may be seen with a hyperechoic center and hypoechoic periphery, corresponding to a fibrocollagenous region centrally and a myxomatous region peripherally [5]. Sonography, however, cannot reliably distinguish benign from malignant lesions [5].

Similarly, neurofibromas can simulate fluid collections on CT scan due to their low attenuation [2]. This has been attributed to myelin lipid content, fat entrapment, and high water content in endoneurial myxoid tissue [2]. Heterogeneity with central necrosis is more commonly noted in MPNSTs and, however, may also be seen in ancient schwannomas [2]. Vascularity is variable; however, irregular nodular peripheral enhancement with corkscrew vessels at angiography is suggestive of MPNSTs [2]. Indistinct margins are more frequent in MPNSTs; however, plexiform neurofibromas may also show ill-defined margins [2].

MRI is the most useful imaging modality to characterize tumour extent and suggest neurogenic origin [6] due to its high contrast resolution and multiplanar capabilities. PNSTs typically show homogenously hyperintense T2 signal or the characteristic target sign with a central hypointense region, oriented longitudinally in the nerve distribution [7]. Wasa et al. showed that the presence of two or more MRI features suggestive of malignancy indicated MPNST with 61% sensitivity and 90% specificity [8]. These features were the presence of a peripheral enhancement pattern, perilesional edema-like zone, intratumoural cystic lesions, and largest dimension of the mass (greater than five centimetres) [8]. Heterogeneity on T1-weighted images was also useful in NF1 patients [8]. Most neurofibromas did not show the target sign; hence, its absence was not a useful discriminator for malignancy [8].

Bensaid et al. showed a 100% negative predictive value (NPV) for malignancy using FDG-PET scans in NF1 patients with symptomatic lesions [9]. Specificity was 86%, meaning that there would be false-positive scans; however, the NPV provides increased confidence in diagnosis of benign tumours when PET scan is negative [9]. PET/CT also proved useful in biopsy planning where the most metabolically active area, reflecting the highest grade of tumour, can be biopsied [1, 10].

Cross-sectional imaging is therefore helpful in the diagnosis and delineation of tumour extent of PNSTs. The target sign can be seen on multiple modalities, however may not always be present, hence demonstrating parent nerve continuity is more useful in diagnosis. Education of NF1 patients on MPNST clinical features is essential for early identification of malignant change and hence improved outcomes [10]. Annual clinical examination in specialized multidisciplinary centres is also recommended for NF1 patients [11]. Detection of MPNSTs using clinical characteristics and CT or MRI alone is extremely difficult [1] due to overlapping signs in benign and malignant lesions. Hence in patients with clinically suspicious PNSTs, PET scan and PET/CT can be useful in deciding further management and directing biopsy.
